# Loss of PRSS56 function leads to ocular angle defects and increased susceptibility to high intraocular pressure

**DOI:** 10.1242/dmm.042853

**Published:** 2020-05-29

**Authors:** Cassandre Labelle-Dumais, Goutham Pyatla, Seyyedhassan Paylakhi, Nicholas G. Tolman, Syed Hameed, Yusef Seymens, Eric Dang, Anil K. Mandal, Sirisha Senthil, Rohit C. Khanna, Meha Kabra, Inderjeet Kaur, Simon W. M. John, Subhabrata Chakrabarti, K. Saidas Nair

**Affiliations:** 1 Department of Ophthalmology, University of California, San Francisco, CA 94143, USA; 2 Brien Holden Eye Research Centre, L V Prasad Eye Institute, Hyderabad 500034, India; 3 Manipal Academy of Higher Education, Manipal, Karnataka 576104, India; 4 Howard Hughes Medical Institute, The Jackson Laboratory, Bar Harbor, ME 04609, USA; 5 Jasti V. Ramanamma Children's Eye Care Centre, L V Prasad Eye Institute, Hyderabad 500034, India; 6 Gullapalli Pratibha Rao International Centre for Advancement of Rural Eye Care, L V Prasad Eye Institute, Hyderabad 500034, India; 7 Department of Anatomy, University of California, San Francisco, CA 94143, USA

**Keywords:** Angle-closure glaucoma, Intraocular pressure, Ocular drainage defects, PRSS56

## Abstract

Glaucoma is a leading cause of blindness, affecting up to 70 million people worldwide. High intraocular pressure (IOP) is a major risk factor for glaucoma. It is well established that inefficient aqueous humor (AqH) outflow resulting from structural or functional alterations in ocular drainage tissues causes high IOP, but the genes and pathways involved are poorly understood. We previously demonstrated that mutations in the gene encoding the serine protease PRSS56 induces ocular angle closure and high IOP in mice and identified reduced ocular axial length as a potential contributing factor. Here, we show that *Prss56^−/−^* mice also exhibit an abnormal iridocorneal angle configuration characterized by a posterior shift of ocular drainage structures relative to the ciliary body and iris. Notably, we show that retina-derived PRSS56 is required between postnatal days 13 and 18 for proper iridocorneal configuration and that abnormal positioning of the ocular drainage tissues is not dependent on ocular size reduction in *Prss56^−/−^* mice. Furthermore, we demonstrate that the genetic context modulates the severity of IOP elevation in *Prss56* mutant mice and describe a progressive degeneration of ocular drainage tissues that likely contributes to the exacerbation of the high IOP phenotype observed on the C3H/HeJ genetic background. Finally, we identify five rare *PRSS56* variants associated with human primary congenital glaucoma, a condition characterized by abnormal development of the ocular drainage structures. Collectively, our findings point to a role for PRSS56 in the development and maintenance of ocular drainage tissues and IOP homeostasis, and provide new insights into glaucoma pathogenesis.

## Abstract (precis)

**Summary:** This study reports a novel role of PRSS56 in the proper developmental positioning of ocular drainage tissues, establishing it as an important genetic factor involved in iridocorneal angle configuration and intraocular pressure homeostasis.

## INTRODUCTION

Glaucoma is a leading cause of blindness and refers to a heterogeneous group of diseases characterized by progressive loss of retinal ganglion cells (RGCs) and corresponding visual field defects. One of the major risk factors for developing glaucoma is elevated intraocular pressure (IOP) (Quigley, 1996). Regulation of IOP mainly relies on the balance between aqueous humor (AqH) production in the ciliary body and its exit through ocular drainage structures located at the iridocorneal angle that forms at the junction between the iris and the cornea (Moses, 1987; Shields, 1992). The key ocular drainage structures consist of the trabecular meshwork (TM) and the Schlemm's canal. AqH enters the TM and subsequently drains into the Schlemm's canal before entering the collector channels, which culminate in the episcleral veins (Vranka et al., 2015; Carreon et al., 2017). Consistent with a critical role for ocular drainage tissues in AqH outflow regulation, structural or functional alterations of these tissues are well-established causes of high IOP (Shields, 1982a,b).

Based on the configuration of the iridocorneal angle and age of onset, glaucoma is broadly divided into primary open angle glaucoma (POAG), primary angle-closure glaucoma (PACG) and primary congenital glaucoma (PCG) (Shields, 1982c). In POAG, the ocular angle structures are open, providing AqH direct access to the drainage tissues. Despite no obvious obstruction of the iridocorneal angle in POAG, AqH outflow is impaired leading to elevated IOP. In PACG, altered positioning of the peripheral iris causes blockade and closure of the iridocorneal angle preventing access of AqH to the drainage tissues (Shields, 1982b; Ritch and Lowe, 1996; Ritch et al., 1996). Anatomical features such as reduced ocular size, relatively larger lens and a shallow anterior chamber predispose the eye to pupillary block, which restricts the flow of AqH from the posterior to the anterior chamber (Quigley, 2009; Lowe, 1970). The accumulation of AqH in the posterior chamber causes the iris to bulge anteriorly, leading to its apposition against the ocular drainage tissues (angle-closure). Genome-wide association studies (GWAS) and family-based linkage analyses have implicated variants of multiple genes in adult and juvenile forms of POAG, respectively (Wiggs and Pasquale, 2017; Liu and Allingham, 2017). Although no major genes have been linked to PACG, GWAS studies have found population-specific associations for a few genes (Aung and Khor, 2016). Primary congenital glaucoma (PCG) is a neonatal or early infancy autosomal recessive disease characterized by abnormal development of ocular angle structures. Collectively, mutations in *CYP1B1*, *LTBP2*, *MYOC*, *FOXC1* and *GPATCH3* constitute a major cause of PCG worldwide (Cascella et al., 2015; Lewis et al., 2017; Chakrabarti et al., 2009; Vasiliou and Gonzalez, 2008; Kaur et al., 2005; Miller et al., 2017; Wiggs, 2015; Craig and Mackey, 1999). However, mutations in these genes account for less than 50% of all PCG cases, suggesting that additional genes contribute to the etiology of PCG. Notably, recent studies have also implicated autosomal dominant mutations in the genes coding for the endothelial cell receptor TEK and its ligand angiopoietin 1 (ANGPT1) (Souma et al., 2016; Thomson et al., 2017), and identified genetic and physical interactions between *CYP1B1* and *TEK* in PCG (Kabra et al., 2017).

We, and others, have previously demonstrated that mutations in the gene coding for the secreted serine protease PRSS56 result in a reduction in ocular axial length (nanophthalmos) in humans, and many of these individuals go on to develop angle-closure glaucoma (ACG) (Nair et al., 2011; Gal et al., 2011; Orr et al., 2011). Consistent with the clinical manifestations observed in humans, mice homozygous for a *Prss56* mutation (*Prss56^glcr4^*, previously referred to as *Prss56^Grm4^*) exhibit reduced ocular axial length and develop glaucomatous neurodegeneration and other pathophysiological hallmarks of ACG (Nair et al., 2011). Of note, their lens diameter is indistinguishable from that of the control eyes, causing the lens to occupy a relatively larger ocular volume (Nair et al., 2011; Paylakhi et al., 2018). As both ocular size reduction and a relatively larger lens predispose to angle closure, we have previously proposed that these anatomical features could cause physical blockage of the drainage tissues (ocular angle closure) leading to high IOP in *Prss56* mutant mice (Nair et al., 2011). Consistent with this, AqH outflow was significantly compromised in *Prss56* mutant eyes, supporting the proposal that inefficient AqH drainage contributes to IOP elevation. However, it is unclear whether the angle-closure and high IOP phenotypes caused by *Prss56* mutations are a direct consequence of ocular size reduction or whether other factors that have yet to be characterized are participating.

Here, we use a combination of genetic, histological and physiological approaches to determine the role of PRSS56 in the development of ocular drainage tissues, characterize the iridocorneal angle defects resulting from loss of PRSS56 function and evaluate their contribution to the high IOP phenotype observed in *Prss56* mutant mice. In addition, we performed a genetic screen to test for the potential involvement of *PRSS56* mutations in PCG. Overall, our findings provide new insight into the role of PRSS56 in the development and maintenance of ocular drainage structures and pathogenic processes underlying iridocorneal angle defects and high IOP caused by *PRSS56* mutations.

## RESULTS

### Loss of PRSS56 function leads to altered iridocorneal configuration and high IOP

**Fig. 1.**
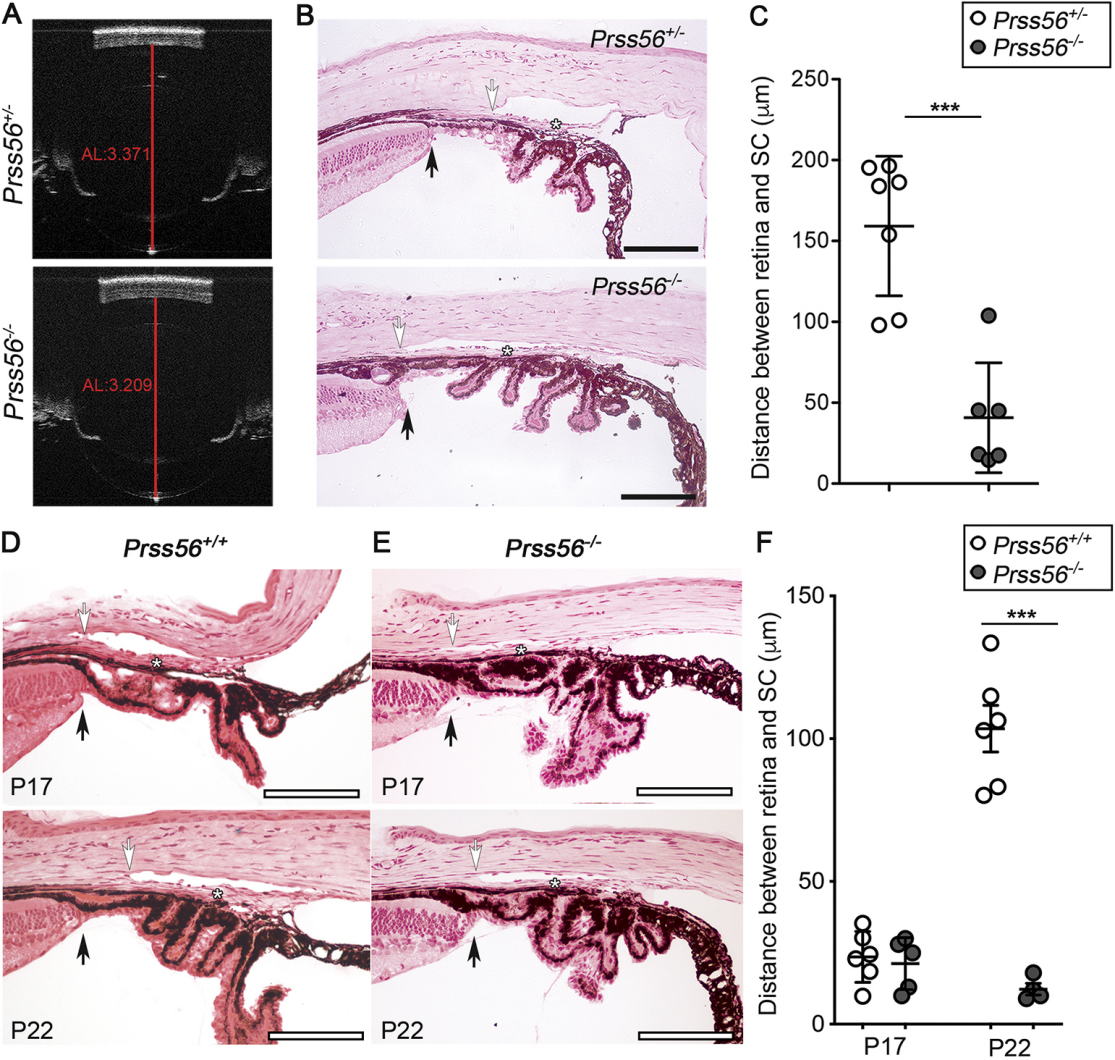
***Prss56* knockout mice show altered positioning of ocular angle structures.** (A) Representative OCT images showing reduced ocular size in *Prss56^−/−^* compared to *Prss56^+/−^* mice as previously described (Paylakhi et al., 2018). Shown are eyes from 2-month-old mice. The red lines indicate ocular axial length (AL). (B) Representative H&E stained ocular sections from *Prss56^−/−^* and control *Prss56^+/−^* mice showing altered organization of ocular angle structures in *Prss56^−/−^* mice that is characterized by a posterior shift in the position of the TM (asterisks) and adjoining Schlemm's canal (white arrows) relative to the iris and ciliary body, resulting in angle-closure. Black arrows indicate peripheral retina. (C) The distance between the peripheral retina and the posterior edge of the Schlemm's canal is significantly reduced in *Prss56^−/−^* eyes compared to control *Prss56^+/−^* eyes. (D,E) Representative H&E stained ocular sections from *Prss56^+/+^* (D) and *Prss56^−/−^* (E) mice at different postnatal developmental stages showing that the distance between the peripheral edge of the retina (black arrows) and the posterior edge of the Schlemm's canal (white arrows) is narrow at P17 and is markedly increased at later time points (P22) in *Prss56^+/+^*, but not in *Prss56^−/−^* mice, leading to an anterior positional shift of ocular drainage tissues and open angle configuration. Asterisks indicate position of the TM. In C, *n*=7 *Prss56^+/−^* eyes, *n*=6 *Prss56^−/−^* eyes. In F, *n*=6 P17 *Prss56^+/+^* eyes, *n*=5 P17 *Prss56^−/−^* eyes, *n*=6 P22 *Prss56^+/+^* eyes, *n*=4 P22 *Prss56^−/−^* eyes. Data are mean±s.d., ***P<0.001, Student's *t*-test. Scale bars: 100 µm.

**Fig. 2.**
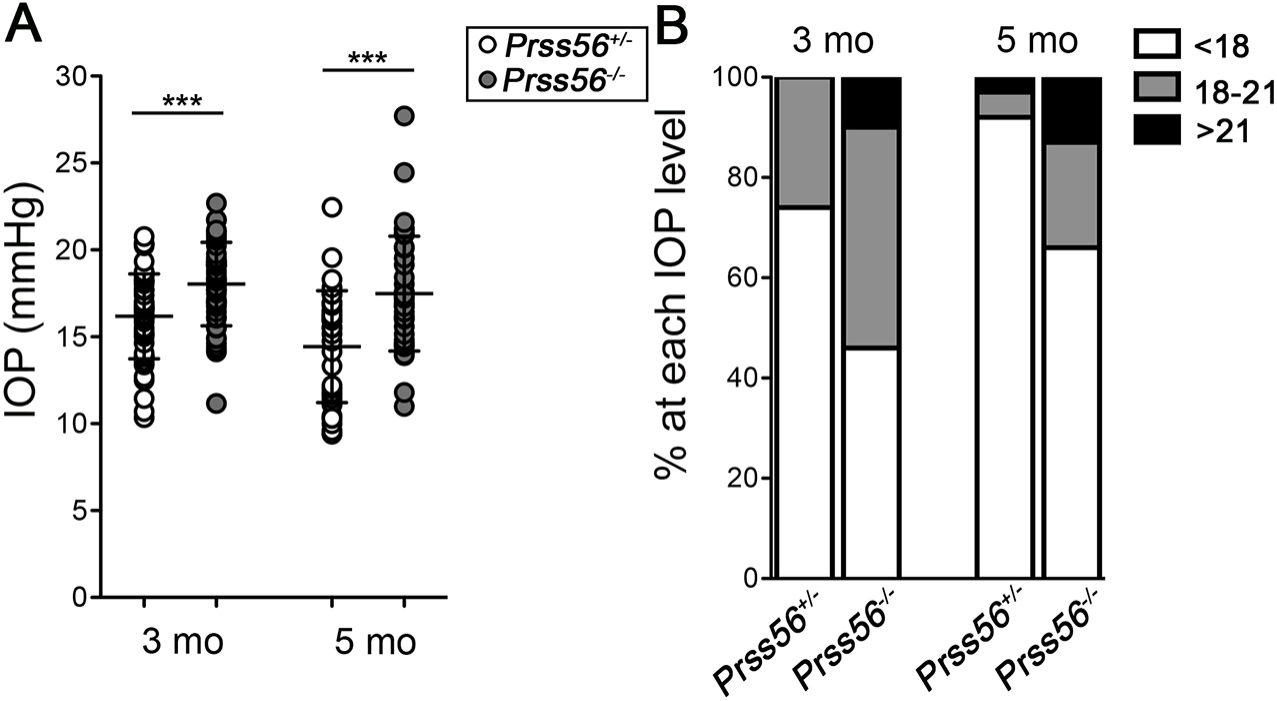
***Prss56* knockout mice develop elevated IOP.** (A) IOP is significantly higher in *Prss56^−/−^* mice compared to control *Prss56^+/−^* mice at 3 and 5 months (mo). *n*=50 and *n*=48 for *Prss56^+/−^* eyes at 3 and 5 months, respectively; *n*=38 *Prss56^−/−^* eyes at each age examined. Data are mean±s.d., ***P<0.001, Student's *t*-test. (B) Histogram showing the proportion of eyes in each IOP range at 3 and 5 months.

The *Prss56^glcr4^* mutation we originally characterized in the mouse leads to a truncated PRSS56 protein with an intact catalytic domain (Nair et al., 2011), raising the question whether the ocular phenotypes observed in *Prss56^glcr4/glcr4^* mice result from a loss or gain of PRSS56 function. To address this, we recently used mice homozygous for a null allele of *Prss56* (*Prss56^−/−^*) to demonstrate that loss of PRSS56 function causes ocular axial length reduction, an anatomical feature predisposing to ACG (Fig. 1A) (Paylakhi et al., 2018). To further define the ocular phenotype resulting from loss of PRSS56 function, we performed a detailed histological analysis of ocular angle structures in *Prss56^−/−^* mice. Because *Prss56^+/−^* eyes are indistinguishable from *Prss56^+/+^* eyes, they were used as controls for all experiments described in this study unless otherwise specified (Paylakhi et al., 2018). In control adult eyes, the drainage structures (TM and Schlemm's canal) are located anterior to the very peripheral angle recess (which is populated by the iris root and ciliary body), allowing AqH to freely access the ocular drainage tissues without obstruction by the iris (Fig. 1B). In contrast, the TM and Schlemm's canal of age-matched *Prss56^−/−^* eyes were found in close proximity of the peripheral retina and exhibit a posterior shift in their position relative to the ciliary body and iris (Fig. 1B). Consistent with this observation, the distance between the edge of the peripheral retina and the posterior end of the Schlemm's canal was significantly smaller in *Prss56^−/−^* eyes compared to *Prss56^+/−^* eyes (Fig. 1C). Interestingly, histological analysis of wild-type eyes revealed that the drainage structures undergo an anterior positional shift during normal postnatal ocular development (compare the position of the Schlemm's canal relative to the ciliary body and iris at P17 and P22 in Fig. 1D). In contrast, no anterior shift in the position of the ocular drainage tissues was observed in *Prss56^−/−^* mice (Fig. 1E). Accordingly, the distance between the edge of the peripheral retina and the posterior end of the drainage structures increases during ocular development in wild-type but not in *Prss56^−/−^* mice (Fig. 1F). These findings suggest that PRSS56 deficiency impairs the anterior positional shift of the drainage structures, predisposing *Prss56^−/−^* eyes to physical obstruction of the iridocorneal angle by the peripheral iris (Fig. 1B and bottom panel of Fig. 1E). Supporting this, *Prss56^−/−^* mice were more susceptible to develop high IOP compared to their *Prss56^+/−^* littermates at both ages examined (3 and 5 months) (Fig. 2). Of note, beside ocular axial length reduction and iridocorneal angle defects, *Prss56^−/−^* eyes did not exhibit any gross morphological abnormalities compared to control eyes (Fig. S1). Collectively, these findings demonstrate that, in addition to causing ocular size reduction, loss of PRSS56 function leads to altered positioning of the TM and Schlemm's canal, an anatomical feature that could contribute to a variety of ocular angle-related defects, including an increased susceptibility to develop angle closure and impaired IOP homeostasis.

### Spatiotemporal requirement of PRSS56 for proper configuration of the iridocorneal angle

**Fig. 3.**
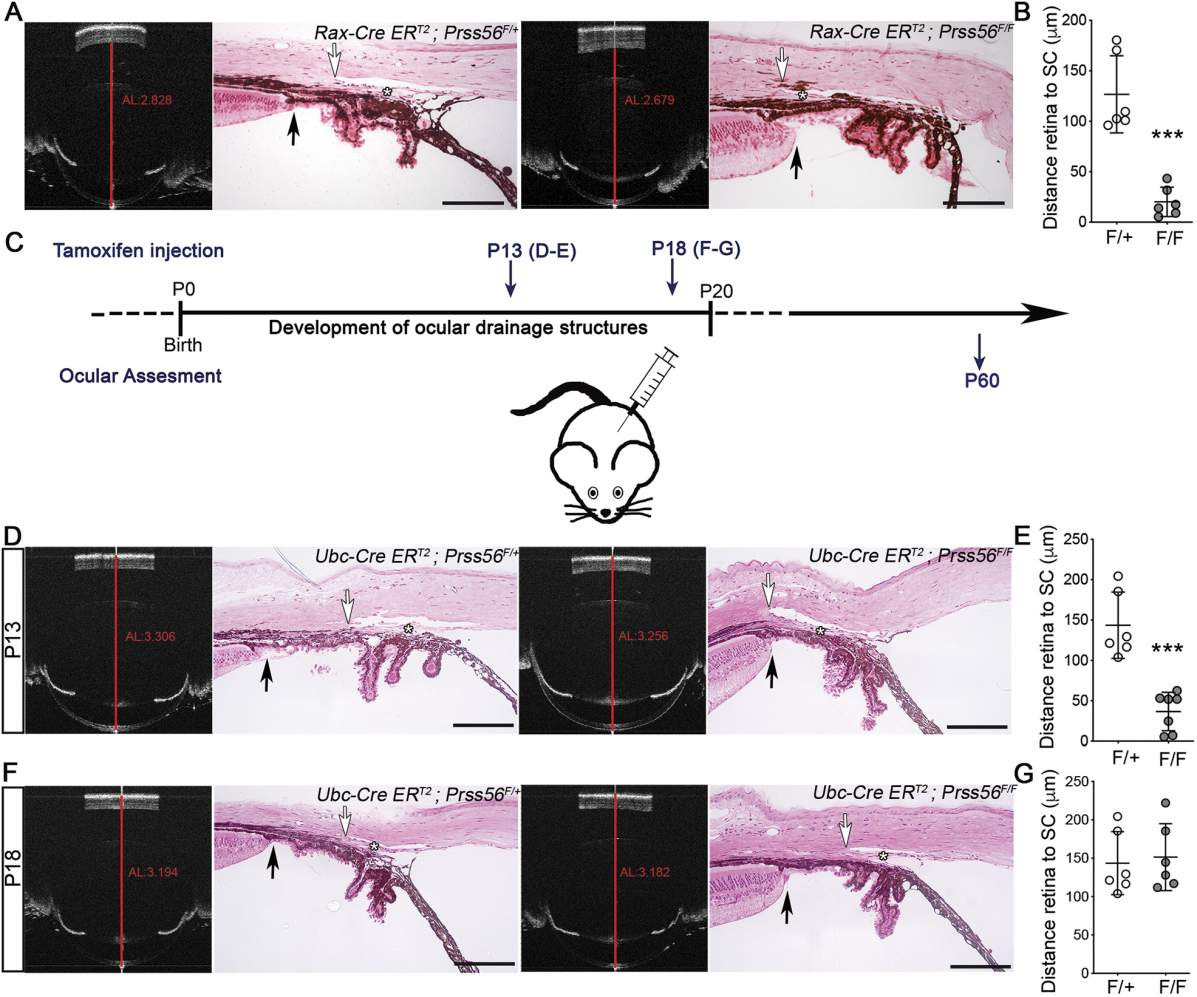
**Spatiotemporal requirement of PRSS56 for proper positioning of ocular angle structures.** (A,B) Representative OCT images and H&E-stained ocular sections (A) from control *Rax*-*Cre^ERT2^;Prss56^F/+^* and *Rax-Cre^ERT2^;Prss56^F/F^* mice following tamoxifen injection at P8, a time point that coincides with the final differentiation of Müller cells. As described previously (Paylakhi et al., 2018), *Rax-Cre^ERT2^;Prss56^F/F^* mice (F/F) show reduced ocular axial length (AL, red lines on OCT images) compared to control *Rax*-*Cre^ERT2^;Prss56^F/+^* mice (F/+). In addition, *Rax-Cre^ERT2^;Prss56^F/F^* mice exhibit a posterior shift in the position of the TM (asterisk) and adjoining Schlemm's canal relative to the iris and ciliary body, leading to a shorter distance between the peripheral retina (black arrows) and the posterior edge of the Schlemm's canal (white arrows) (quantified in B) and angle closure. (C) Schematic illustrating the tamoxifen injection paradigm for conditional inactivation of *Prss56* at P13 and P18. (D,E) Representative OCT images and H&E-stained ocular sections (D) showing reduced ocular axial length as previously described (Paylakhi et al., 2018), and abnormal organization of ocular angle structures characterized by a posterior shift in the position of the TM (asterisk) and adjoining Schlemm's canal relative to the iris and ciliary body, leading to a shorter distance between the peripheral retina (black arrows) and the posterior edge of the Schlemm's canal (white arrows) (quantified in E) and angle closure in *Ubc-Cre^ERT2^;Prss56^F/F^* mice (F/F) following tamoxifen injection at P13 compared to control *Ubc*-*Cre^ERT2^;Prss56^F/+^* mice (F/+). (F,G) In contrast, only a marginal decrease in ocular axial length (OCT images) and no alteration in the distance between the peripheral retina (black arrows) and the posterior edge of the Schlemm's canal (white arrows) (quantified in G) or ocular angle structures organization (as shown by an open angle configuration) were observed in *Ubc-Cre^ERT2^;Prss56^F/F^* mice following tamoxifen injection at P18 compared to control eyes. *n*=6-7 eyes/group. As control eyes (F/+) injected with tamoxifen at the earliest time point (P13) did not show any effect on the positioning of ocular drainage tissues, they were used as controls in both E and G. Data are mean±s.d., ***P<0.001, Student's *t*-test. Scale bars: 100 µm.

To determine the spatiotemporal requirement of PRSS56 function for proper positioning of ocular angle structures, we used the conditional *Prss56* mutant mouse model we validated and characterized recently to ablate *Prss56* in a tissue-specific manner or at distinct stages of ocular development (Paylakhi et al., 2018). We have previously shown that *Prss56* ocular expression is restricted to the retina and not detected in the anterior segment (Paylakhi et al., 2018). Specifically, *Prss56* expression is predominantly observed in a subset of Müller cells. To confirm the role of retina-derived PRSS56 in the configuration of the iridocorneal angle, we crossed mice carrying the conditional floxed *Prss56* allele (*Prss56^F^*) to the *Rax-CreER^T2^* line to selectively ablate *Prss56* from Müller glia. In addition to the reduction in ocular axial length that we described previously (Paylakhi et al., 2018), we detected a posterior shift in the positioning of ocular drainage structures in *Rax-Cre^ERT2^;Prss56^F/F^* mice similar to that observed in *Prss56*^−/−^ mice, clearly establishing a requirement for Müller glia-derived PRSS56 in the configuration of the iridocorneal angle (Fig. 3A,B). To determine the temporal requirement of PRSS56 function for proper positioning of ocular angle structures, we next bred conditional *Prss56* mutant mice to the inducible ubiquitous *Ubc-CreER^T2^* mouse line to ablate *Prss56* at distinct developmental stages. Conditional *Prss56* mutant mice carrying the inducible *Ubc-Cre* transgene (*Ubc-Cre^ERT2^;Prss56^F/F^*) and control (*Ubc*-*Cre^ERT2^;Prss56^F/+^*) mice were injected with tamoxifen at postnatal day (P) 13 or P18 (Fig. 3C), to selectively inactivate *Prss56* when ocular drainage structures are actively developing or when iridocorneal angle morphogenesis is mostly complete, respectively (Kizhatil et al., 2014). Histological analysis of P60 eyes revealed an open-angle configuration in control mice injected at either time points (Fig. 3D,F). In contrast, eyes from *Ubc-Cre^ERT2^;Prss56^F/F^* mice injected at P13, but not P18, displayed ocular axial length reduction, as described previously (Paylakhi et al., 2018), as well as an altered iridocorneal configuration characterized by a posterior shift in the positioning of ocular drainage structures similar to the ocular phenotype observed in *Prss56*^−/−^ mice (Fig. 3D-G). Together, these findings indicate that PRSS56 produced by Müller glia is required between P13 and P18 for proper iridocorneal angle configuration.

### Independent roles for PRSS56 in ocular size determination and iridocorneal angle configuration

**Fig. 4.**
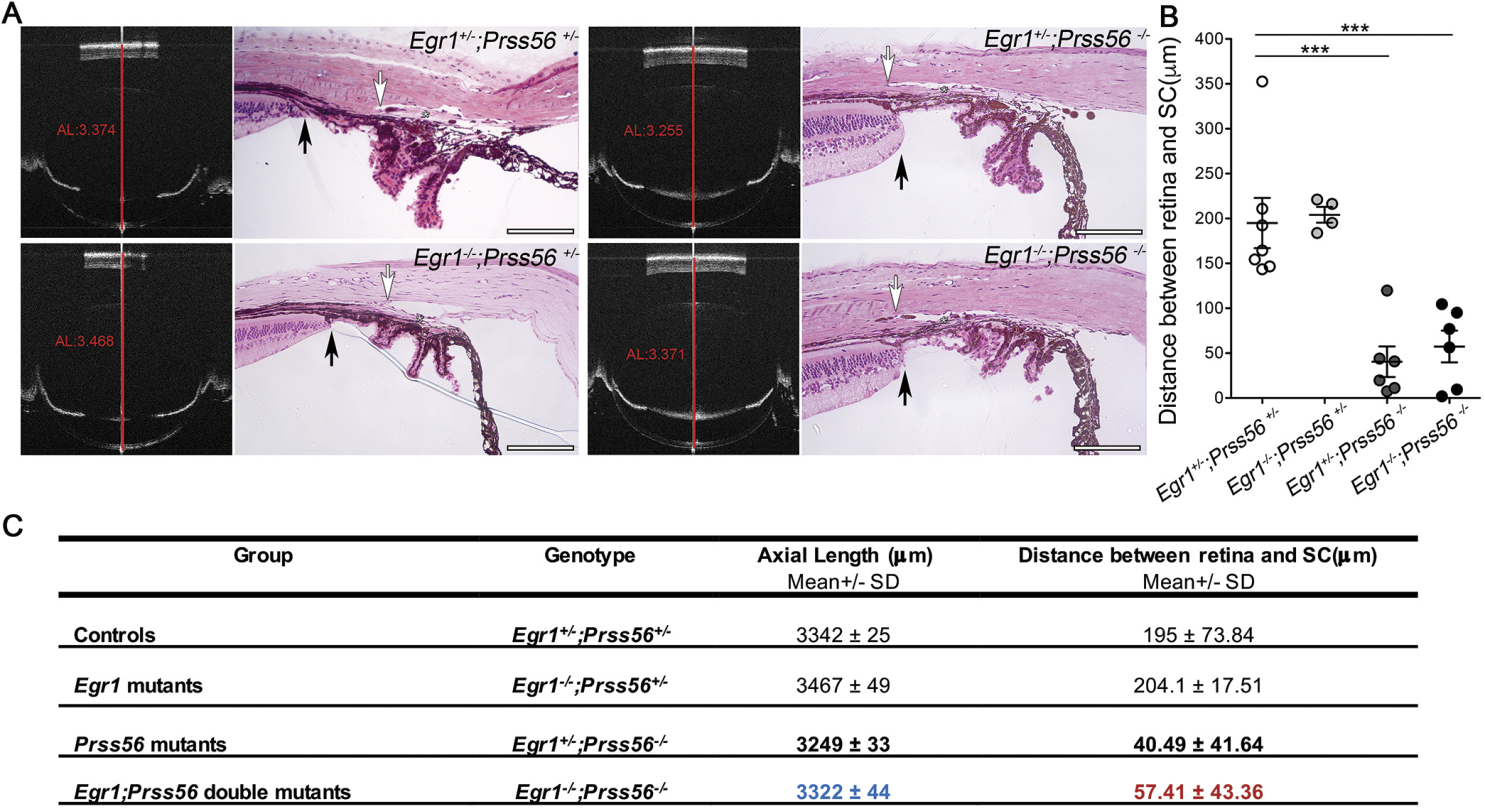
**Altered positioning of ocular angle structures is independent of axial length reduction in *Prss56* mutant mice.** (A) Representative OCT images and H&E-stained ocular sections demonstrating that *Egr1* inactivation rescues ocular size reduction but not the altered organization of ocular angle structures in *Prss56^−/−^* mice. The red lines on OCT images indicate ocular axial length (AL). Compared to control *Egr1^+/−^;Prss56^+/−^* eyes, *Prss56* mutants (*Egr1^+/−^;Prss56^−/−^*) exhibit reduced ocular axial length and a posterior shift in the position of ocular drainage structures (asterisks) relative to the iris and ciliary body resulting in angle closure. In contrast, in *Egr1;Prss56* double mutant mice (*Egr1^−/−^;Prss56^−/−)^*, the ocular axial length is indistinguishable from that of control mice (*Egr1^+/−^;Prss56^+/−^*) (previously described in Paylakhi et al., 2018), whereas ocular drainage tissues exhibit a posterior shift relative to the iris and ciliary body similar to that observed in *Prss56* mutant mice (*Egr1^+/−^;Prss56^−/−^*). These data demonstrate that *Egr1* inactivation rescues ocular axial length reduction (compare value in blue to bold value above in C) but not iridocorneal configuration defects and angle-closure phenotype (compare value in red to bold value above in C) in *Prss56* mutant mice (shown are 2-month-old eyes). (B) Quantification of the distance between the peripheral edge of the retina and posterior end of the Schlemm's canal (SC) showing a significant reduction in both *Egr1^+/−^;Prss56^−/−^* and *Egr1^−/−^;Prss56^−/−^* eyes compared to control *Egr1^+/−^;Prss56^+/−^* eyes. *n*=7 control (*Egr1^+/−^;Prss56^+/−^*), *n*=4 *Prss56* mutants (*Egr1^+/−^;Prss56^−/−^*), *n*=6 *Egr1* mutant (*Egr1^−/−^;Prss56^+/−^*), *n*=6 double mutant (*Egr1^−/−^;Prss56^−/−^*) eyes. Data are mean±s.e.m. ***P<0.001, one-way ANOVA. (C) Summary table showing the mean values for ocular axial length and the distance between the retina and Schlemm's canal for each genotype. Scale bars: 100 µm.

**Fig. 5.**
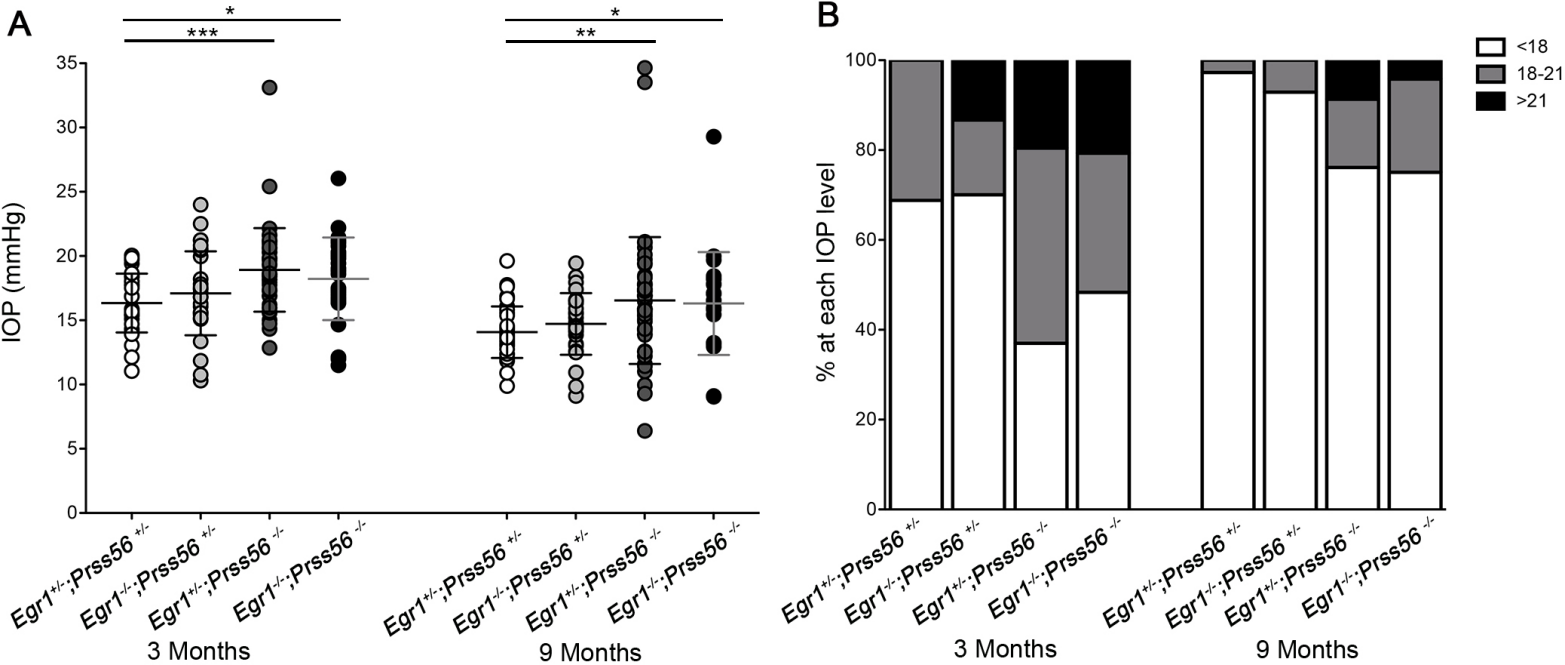
**Genetic rescue of ocular size reduction does n**o**t prevent IOP elevation in *Prss56* mutant mice.** (A) Scatter plot showing IOP values at 3 and 9 months old. IOP values of *Egr1* single mutant (*Egr1^−/−^;Prss56^+/−^*) mice were not significantly different from those of control mice (*Egr1^+/−^;Prss56^+/−^*). However, IOP was significantly higher in *Prss56* single mutant (*Egr1^+/−^;Prss56^−/−^*) and *Egr1^−/−^;Prss56^−/−^* mice compared to the control (*Egr1^+/−^;Prss56^+/−^*) mice at 3 and 9 months. *n*=32 and 36 for control (*Egr1^+/−^;Prss56^+/−^*) eyes at 3 and 9 months, respectively; *n*=30 and 28 for *Egr1* single mutant (*Egr1^−/−^;Prss56^+/−^*) eyes at 3 and 9 months, respectively; *n*=46 for *Prss56* single mutant (*Egr1^+/−^;Prss56*^−/−^) at each age examined; *n*=29 and 24 for *Egr1^−/−^;Prss56^−/−^* eyes at 3 and 9 months, respectively. Data are mean±s.d. *P<0.05, **P<0.01, ***P<0.001, one-way ANOVA. (B) Histogram showing the proportion of eyes in each IOP range at 3 and 9 months.

Because reduced ocular size is an anatomical feature predisposing to ACG, we wanted to determine whether the ocular axial length reduction we have previously described in *Prss56^−/−^* mice is responsible for the altered positioning of ocular drainage structures (Paylakhi et al., 2018). To test this possibility, we took advantage of the *Egr1;Prss56* double mutant (*Egr1^−/−^;Prss56^−/−^*) mouse model we have recently generated and characterized (Paylakhi et al., 2018). In these mice, inactivation of *Egr1* was found to rescue the ocular axial length reduction caused by loss of PRSS56 function. Because the ocular axial length in *Egr1^−/−^;Prss56^−/−^* mice is similar to that of control mice (Fig. 4A,C), it allows us to test whether ocular axial length reduction contributes to the altered positioning of drainage structures in *Prss56^−/−^* mice. We have previously shown that *Egr1^+/−^;Prss56^+/−^* eyes are indistinguishable from wild-type (*Egr1^+/+^;Prss56^+/+^*) eyes, hence they were used as controls (Paylakhi et al., 2018). Although both *Egr1^−/−^;Prss56^+/−^* and control *Egr1^+/−^;Prss56^+/−^* eyes displayed an open iridocorneal angle configuration, histological analysis of *Egr1^−/−^;Prss56^−/−^* eyes revealed a posterior positional shift of ocular drainage structures relative to the ciliary body and iris, a phenotype reminiscent of that observed in *Prss56^−/−^* and *Egr1^+/−^;Prss56^−/−^* mice (Figs 1B and 4A). In agreement with this observation, the distance between the edge of the peripheral retina and the posterior end of the Schlemm's canal in *Egr1^−/−^;Prss56^−/−^* eyes was significantly smaller than that of control *Egr1^+/−^;Prss56^+/−^* eyes and comparable to that of *Prss56^−/−^* and *Egr1^+/−^;Prss56^−/−^* eyes (Figs 1C and 4B,C). In addition, and consistent with the observed ocular angle defects, *Egr1^−/−^;Prss56^−/−^* mice were more susceptible to developing high IOP compared to control *Egr1^+/−^;Prss56^+/−^* mice (Fig. 5). Together, these results demonstrate that *Egr1* inactivation rescues ocular axial length reduction in *Prss56* mutant mice but not the altered positioning of ocular drainage structures. Furthermore, these findings suggest that the altered iridocorneal angle configuration resulting from loss of PRSS56 function can occur independently from ocular size reduction and that interacting genetic factors can selectively modulate PRSS56 functions.

### The severity of IOP elevation in *Prss56* mutant mice is modulated by the genetic context

**Fig. 6.**
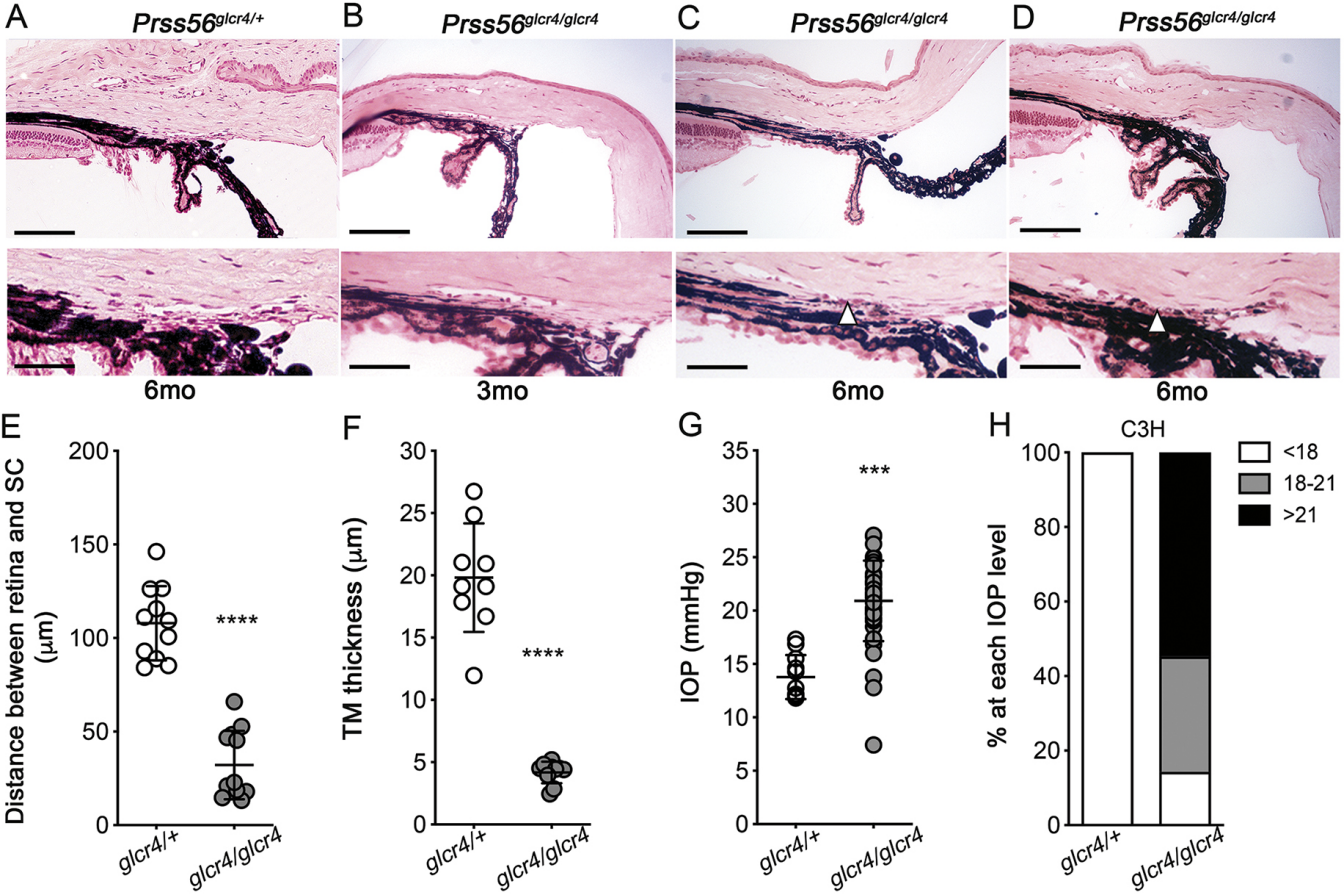
**The IOP elevation and ocular drainage structure abnormalities observed in *Prss56* mutant mice are genetic context-dependent.** (A-D) Representative H&E-stained ocular sections from control C3H.*Prss56^glcr4/+^* (A) and C3H.*Prss56^glcr4/glcr4^* mutant (B-D) mice maintained on a C3H/HeJ genetic background showing that by 6 months of age, *Prss56* mutant mice exhibit a hypoplastic TM layer (arrowhead in C and D). (E) Scatter plot showing significant reduction in the distance between the retina and Schlemm's canal in *Prss56* mutant mice (*Prss56^glcr4/glcr4^*) compared to their control littermates (*Prss56^glcr4/+^*). *n*=11 and 12 eyes for control and mutant mice, respectively. (F) Scatter plot showing TM thickness in 6-month-old *Prss56* mutant mice (*Prss56^glcr4/glcr4^*) compared to their control littermates (*Prss56^glcr4/+^*). *n*=9-12 eyes/group. (G,H) Scatter plot showing IOP values (G) and histogram showing the proportion of eyes in each IOP range (H) for control (C3H.*Prss56^glcr4/+^*) and *Prss56* mutant (C3H.*Prss56^glcr4/glcr4^*) mice maintained on the C3H/HeJ genetic background aged between 3 and 5 months. IOP values were significantly higher in *Prss56* mutant mice compared to control mice. *n*=9 *Prss56^glcr4/+^* and *n*=10 *Prss56^glcr4/glcr4^* eyes in E; *n*=11 *Prss56^glcr4/+^* and *n*=12 *Prss56^glcr4/glcr4^* eyes in F; *n*=11 *Prss56^glcr4/+^* and *n*=42 *Prss56^glcr4/glcr4^* eyes in G and H. Data are mean±s.d. ***P<0.001, ****P<0.0001, Student's *t*-test. Scale bars: 100 µm (top panels); 40 µm (lower panels).

The genetic context is well established to modulate the susceptibility to develop high IOP (Choquet et al., 2018; Anderson et al., 2006). To study the effect of the genetic context on ocular angle defects and high IOP caused by *Prss56* mutations, we backcrossed a previously characterized *Prss56* mutant mouse model (*Prss56^glcr4^*) (Nair et al., 2011) on a C3H/HeJ background (C3H.*Prss56^glcr4/glcr4^*). In addition to ocular size reduction, histological analysis revealed a posterior shift in the position of ocular drainage tissues relative to the iris and ciliary body and a progressive thinning of the TM layer in C3H.*Prss56^glcr4/glcr4^* eyes (Fig. 6A-F). Consistent with the observed ocular drainage tissue defects, these mice were highly susceptible to developing elevated IOP (Fig. 6G,H). Notably, although control C3H.*Prss56^glcr4/+^* mice did not develop high IOP, the mean IOP value and proportion of C3H.*Prss56^glcr4/glcr4^* mice developing high IOP were substantially greater compared to *Prss56^−/−^* mice maintained on a C57BL/6J background (*B6. Prss56^−/−^*) (mean IOP value of 20.92 mmHg and 17.79 mmHg for C3H.*Prss56^glcr4/glcr4^* and *B6.Prss56^−/−^* mice, respectively; compare Fig. 6G to Fig. 2A and Fig. 6H to Fig. 2B). Collectively, our findings suggest that the genetic context influences the severities of the IOP elevation and iridocorneal defects caused by *Prss56* mutations. Furthermore, the progressive nature of the TM defects suggest that PRSS56 not only plays a role in the developmental configuration of the iridocorneal angle, but also in the maintenance of ocular drainage tissues and IOP homeostasis.

### Identification of rare *PRSS56* variants in PCG patients

**Fig. 7.**
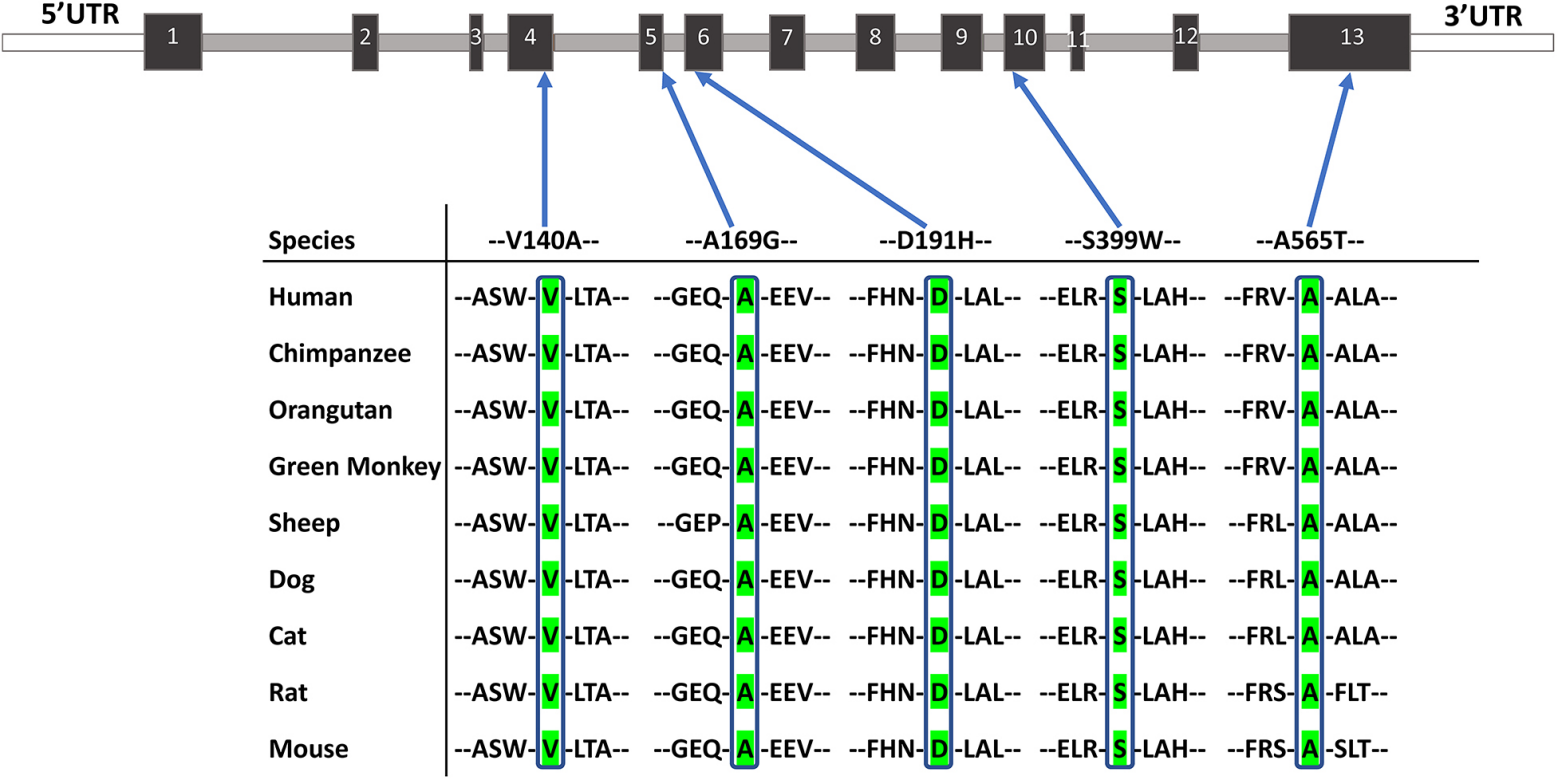
**Identification of rare *PRSS56* variants in PCG patients.** PRSS56 protein sequence alignment across multiple species showing high conservation of amino acid residues for each of the putative *PRSS56* mutations identified in PCG patients.

To determine the relevance of PRSS56 in the development and homeostasis of ocular drainage tissues in humans, we assessed whether *PRSS56* mutations contribute to PCG. Here, we chose to specifically focus on PCG as this form of glaucoma has a strong genetic basis and is characterized by developmental TM and iridocorneal angle defects. To this end, we screened for the presence of rare *PRSS56* variants in a large cohort of classical PCG cases (*n*=316) without homozygous mutations in known PCG-associated genes. We identified 41 *PRSS56* variants, of which 20 were located in coding exons and two in the untranslated regions of two exons, whereas the remaining 19 variants were found to occur in introns (Table S1). Among the coding variants, 11 were non-synonymous missense changes, of which five were predicted to be pathogenic by bioinformatic analysis (Table 1). Notably, all five of these putative pathogenic variants occurred in residues that are highly conserved across species (Fig. 7). Four of these variants were absent in the 1291 control subjects and rarely observed in global populations; one was observed in a control subject (Table S1). The allele frequencies of all other variants were not significantly different between PCG cases and controls and most of them have been reported in multiple populations across the 1000 Genomes and GnomAD databases (Lek et al., 2016; The 1000 Genomes Project Consortium et al., 2010), with frequencies similar to those observed in our control subjects (Table S1).

**Table 1.**
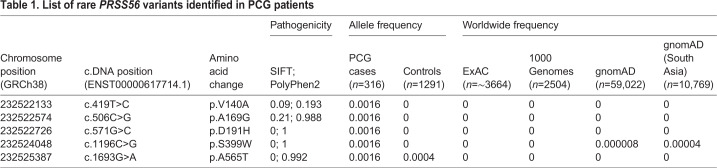
**List of rare *PRSS56* variants identified in PCG patients**

Although PCG is generally considered an autosomal recessive disease (Wiggs and Pasquale, 2017), homozygous *PRSS56* mutations were never observed in these families. However, increasing evidence points to a digenic mode of inheritance for PCG (Schäffer, 2013) and we identified co-inheritance of a heterozygous *CYP1B1* mutation along with a *PRSS56* mutation in two families, PCG152 (*PRSS56*: p.V140A::*CYP1B1*: p.A330T) and PCG279 (*PRSS56*: p.A169G::*CYP1B1*: p.E229K). The parents of these probands harbored either of the allelic variants and were asymptomatic. Moreover, the rare *PRSS56* variants observed in PCG cases were not detected in a POAG cohort (*n*=291). Screening of the entire *PRSS56* gene in the POAG cohort revealed 38 different variants, and the non-synonymous changes were not deemed pathogenic based on bioinformatic analysis and none of these variants was significantly associated with POAG (Table S2).

Among the *PRSS56* intragenic variants identified, an intronic (rs11902035; G allele) and an exonic (rs61744404; C allele) SNP were overrepresented in patients and exhibited strong association (*P*=6.25×10^−12^ and *P*=0.0056, respectively) with PCG that withstood Bonferroni correction (Table S1). As these two polymorphic SNPs spaced at 3.76 kb encompassed all of the five rare *PRSS56* variants identified, we generated intragenic haplotypes in patients harboring these mutations and found that the five patients exhibited the same haplotype (G-C), suggestive of a possible founder effect. Together with findings from our mouse studies, our human data suggest that *PRSS56* mutations may contribute to PCG pathogenesis.

## DISCUSSION

In this study, we use a combination of genetic approaches to show that *Prss56* mutations lead to ocular angle defects in the mouse. We show that, in addition to causing ocular axial length reduction, loss of PRSS56 function from retinal Müller cells leads to an altered configuration of the iridocorneal angle characterized by a posterior shift in the positioning of ocular drainage tissues. Notably, we found that the iridocorneal defects observed in *Prss56* mutant mice are independent from ocular size reduction and described a progressive and genetic context-dependent degeneration of ocular drainage tissues. Finally, we identified five novel rare *PRSS56* variants in PCG patients, two of which were co-inherited with heterozygous *CYP1B1* mutations previously implicated in PCG.

We have previously reported that a mutation in *PRSS56* causes nanophthalmos, a condition characterized by severe ocular axial length reduction (Nair et al., 2011). Nanophthalmic individuals are anatomically predisposed to develop a narrow ocular angle and exhibit a high prevalence of ACG (Nair et al., 2011; Othman et al., 1998). However, not all nanophthalmic individuals go on to develop angle closure, suggesting that reduced ocular size alone is not sufficient to induce physical blockage of the drainage tissues and high IOP (Quigley, 2009). Our data suggest that genetic alterations in factors regulating the positioning of ocular drainage structures may also predispose individuals to angle closure. It is tempting to speculate that natural variation in the anatomical configuration of ocular angle structures in humans has a genetic basis (possibly including *PRSS56* mutations) that could influence the susceptibility to angle closure and high IOP. Supporting this view, the posterior shift in the positioning of TM and Schlemm's canal observed in *Prss56* mutant mice causes the peripheral iris to appear anteriorly displaced in relation to ocular drainage structures, a phenotype reminiscent of Plateau iris configuration (PIC). PIC is characterized by the forward displacement of the ciliary body/pars plana causing an anterior shift in the positioning of the peripheral iris, which can result in occlusion of ocular drainage tissues, impaired AqH outflow and high IOP (Li et al., 2014; Verma et al., 2017). It is conceivable that reciprocal conditions marked by the anterior displacement of the ciliary body and iris or by a posterior shift in the positioning of the drainage tissues could both confer susceptibility to ocular angle closure and impaired IOP homeostasis. Overall, our data suggest that PRSS56 is a critical component of a developmental program regulating iridocorneal angle configuration and that alterations in this process may constitute a risk factor for high IOP and glaucoma.

To address whether ocular axial length reduction contributes to the angle-closure and high IOP phenotypes observed in *Prss56* mutant mice, we took advantage of the *Egr1^−/−^;Prss56^−/−^* genetic mouse model we described recently (Paylakhi et al., 2018). EGR1 is a known regulator of ocular growth (Schippert et al., 2007; Fischer et al., 1999; Ashby et al., 2014) and we show that although *Egr1* inactivation substantially rescues ocular size reduction in *Prss56* mutant mice (Paylakhi et al., 2018), it does not influence the angle-closure and high IOP phenotypes. These findings suggest that the role of PRSS56 in ocular size determination is independent from its role in iridocorneal angle configuration and that PRSS56 could modulate the risk of developing elevated IOP by regulating distinct ocular developmental processes. However, we cannot fully rule out the possibility that the effects of *Prss56* and *Egr1* mutations occur at distinct developmental stages, such that the impact of *Prss56* mutations on ocular axial growth could precede that of *Egr1* inactivation and contribute to altered positioning of ocular angle structures before the observed ocular size rescue. However, this scenario is unlikely as we have previously demonstrated temporal overlap for EGR1 and PRSS56 functions in ocular size regulation (Paylakhi et al., 2018), and thus, our results suggest that genetic factors can selectively modulate PRSS56 functions.

In addition, we have found that the genetic context influences the severity of the high IOP phenotype observed in *Prss56* mutant mice. We show that the magnitude and frequency of IOP elevation are significantly greater in *Prss56* mutant mice maintained on a C3H/HeJ genetic background compared to those maintained on a C57BL6/J background. Furthermore, we observed a progressive degeneration of ocular drainage tissues in C3H.*Prss56^glcr4/glcr4^* mice, which likely impairs AqH outflow and exacerbates IOP elevation. Of note, functional abnormalities of ocular drainage tissues have been found to co-occur with angle closure in subjects with high IOP (mixed mechanisms glaucoma) (Shields, 1996; Ritch, 1996). These findings highlight the genetic complexity underlying the high IOP phenotype and suggest that distinct pathogenic processes, likely acting in an additive manner, contribute to impaired AqH outflow and IOP elevation caused by *PRSS56* mutations. For example, although on its own the posterior shift in the positioning of ocular drainage tissues might only modestly impact AqH outflow, its effect could be exacerbated by other pathogenic insults, such as morphological or functional abnormalities in ocular drainage tissues, leading to high IOP.

We have previously shown that the ocular expression of *Prss56* is restricted to the retina and predominantly detected in a subset of Müller glia enriched in the peripheral region of the retina (Paylakhi et al., 2018). Here, we use a conditional gene targeting approach to demonstrate that PRSS56 derived from retinal Müller cells is required for the proper positioning of ocular drainage tissues. The formation and organization of ocular drainage structures relies on a series of highly orchestrated events starting as early as P0 in the mouse. By P15-P17, morphologically mature drainage structures are established and marked by the presence of a well-defined Schlemm's canal and prominent TM layer (Kizhatil et al., 2014; Smith et al., 2001). Histological analysis of wild-type eyes revealed that, although TM and Schlemm's canal morphogenesis is mostly complete by P17, a subsequent anterior positional shift of ocular drainage tissues must occur for the iridocorneal angle to adopt an open configuration. Indeed, although the TM and Schlemm's canal were found directly adjacent to the edge of the peripheral retina at P17, an anterior shift in their position was observed by P21, leading to an open angle configuration. The altered positioning of ocular drainages tissues observed in *Prss56^−/−^* mice suggests that PRSS56 function is required for the anterior positional shift of the TM and Schlemm's canal. Furthermore, we show that inducing conditional ablation of *Prss56* by tamoxifen injection at P13 (expected to lead to *Prss56* inactivation by P15) led to impaired positioning of the ocular drainage structures. In contrast, and consistent with the developmental timing of the anterior positional shift in wild-type eyes, inducing *Prss56* ablation by tamoxifen injection at P18 (expected to lead to *Prss56* inactivation by P20) had no effect on the positioning of the drainage tissues. Being a secreted protease, and given the proximity of the peripheral retina to the ocular angle structures, PRSS56 produced by Müller cells could contribute to the developmental configuration and positioning of ocular drainage tissues by modulating extracellular matrix remodeling and/or cell-cell and cell-matrix interactions. Supporting this idea, we previously observed focal changes in the extracellular matrix of the TM in *Prss56* mutant mice (Nair et al., 2011). Alternatively, PRSS56 could directly modulate retinal growth and development, which in turn could influence the iridocorneal configuration. Of note, we have previously shown an increase in retinal thickness in *Prss56* mutant mice, which could be attributed to ocular size reduction preventing proper retinal elongation. However, *Egr1* inactivation completely rescues ocular size reduction in *Prss56* mutant mice, but it only partially rescues retinal thickness and has no effect on iridocorneal configuration. Thus, it is possible that PRSS56 could regulate the positioning of ocular drainage tissues, at least in part, via its effect on retinal growth and development.

Consistent with a role for PRSS56 in the organization of ocular angle structures and IOP homeostasis, we identified rare *PRSS56* variants in a cohort of patients with PCG. PCG has been traditionally viewed as an autosomal recessive disease caused by homozygous or compound heterozygous mutations in a major gene, *CYP1B1*, which account for 30-50% of cases worldwide (Wiggs and Pasquale, 2017; Aung and Khor, 2016; Lewis et al., 2017)*.* However, PCG exhibits allelic heterogeneity involving multiple genes that act in concert to influence disease manifestations. Digenic or multiallelic modes of inheritance have been reported for a broad range of complex genetic diseases, including various ocular conditions (Kabra et al., 2017; Schäffer, 2013), where each of the rare alleles contributes only modestly to disease outcome (Schäffer, 2013). Notably, we have previously demonstrated the co-occurrence of heterozygous *CYP1B1* mutations with heterozygous mutations in *MYOC*, *FOXC1* or *TEK* in PCG patients (Chakrabarti et al., 2009; Kaur et al., 2005; Kabra et al., 2017). Here, we identified five putative heterozygous *PRSS56* mutations in PGC patients with no detectable effect on ocular size. Of note, two of the putative *PRSS56* mutations co-occur with heterozygous *CYP1B1* mutations, further supporting a potential role for *PRSS56* mutations in PCG pathogenesis. In the remaining three PCG cases with heterozygous *PRSS56* mutations, we were unable to find a second mutant allele from our panel of 180 glaucoma-associated candidate genes (see Materials and Methods), suggesting the involvement of additional mutation(s) that are yet to be discovered. As the *PRSS56* variants identified in our PCG cohort are relatively rare and rarely occur in global populations (Table S1), screening of additional larger and ethnically diverse PCG cohorts will be essential to definitely establish a role for *PRSS56* mutations in the etiology of PCG. As multiple genes with varying magnitudes of effect on disease outcome contribute to PCG, functional characterization of the rare *PRSS56* variants would help delineate the underlying pathogenic processes.

In summary, we have uncovered a novel role for PRSS56 in the developmental configuration and maintenance of the iridocorneal angle and demonstrate that altered positioning of ocular drainage tissues resulting from loss of PRSS56 function is likely not dependent on ocular axial length reduction. Collectively, our genetic mouse studies and the identification of rare *PRSS56* variants in PCG patients support a potential role for *PRSS56* mutations in the etiology of ocular angle-related diseases and high IOP. Furthermore, the findings presented in this study provide new insights into ocular angle development that have significant implications for our understanding of glaucoma pathogenesis.

## MATERIALS AND METHODS

### Animals

All experiments were conducted in compliance with protocols approved by the Institutional Animal Care and Use Committee at University of California San Francisco (approval numbers: AN153083 and AN120008) or The Jackson Laboratory's Institutional Animal Care and Use Committee. Animals were given access to food and water *ad libitum* and were housed under controlled conditions including a 12 h light/dark cycle in accordance with the National Institutes of Health guidelines. Both male and female mice were used in all experiments and no differences were observed between sexes, and no samples were excluded from the study.

#### Mouse lines used in this study

*Prss56^Cre^* mutant line: C57BL/6.Cg-*Prss56^tm(cre)^* mice have a targeted *Prss56* mutation in which exon1 was replaced by a sequence coding for CRE recombinase to generate a null allele and induce *Prss56* promoter-driven CRE expression (Jourdon et al., 2016).

*Prss56* conditional mutant line (*Prss56^F^*): C57BL/6.Cg-*Prss56^tm1^/*SjJ mice carry LoxP sites flanking exons 2 to 4 of *Prss56*, which result in a catalytically inactive protease in a CRE-dependent manner.

*Egr1* mutant line: B6;129-*Egr1^tm1Jmi^*/J mice have a targeted mutation of *Egr1* resulting in a null allele (Lee et al., 1995).

*Ubc-Cre ER^T2^* transgenic line: C57BL/6.Cg-Tg(UBC-Cre/ERT2)1Ejb mice express the ubiquitous inducible Ubc-Cre recombinase in response to tamoxifen (Ruzankina et al., 2007). *Ubc-Cre^ERT2^;Prss56^F/F^* mice were injected with tamoxifen at P13 and P18 to selectively inactivate *Prss56* when ocular drainage structures are actively developing or largely developed, respectively.

*Rax-Cre ER^T2^* transgenic line: *Rax-Cre ER^T2^;Rax^tm1.1(cre/ERT2)Sbls^*/J mice express tamoxifen-inducible CRE recombinase under the control of the *Rax* promoter (Pak et al., 2014). *Rax-Cre^ERT2^;Prss56^F/F^* mice were injected with tamoxifen at P8 to ablate *Prss56* at a time point that coincides with the final differentiation of Müller cells.

*Prss56^glcr4^* mutant line: C3H.Cg-*Prss56^glcr4^*/SjJ mice carry an ENU-induced truncation mutation in *Prss56* (leading to PRSS56 protein lacking 104 amino acids from its carboxy-terminus; *Prss56^glcr4^*) that was introduced into the C3H/HeJ (C3H) inbred genetic background and backcrossed for at least 10 generations. C3H mice spontaneously develop retinal degeneration as a result of an endogenous *PDE6b* mutation. Hence, we have used a C3H substrain corrected for the inherent *PDE6b* mutation (C3A.BLiA-*Pde6b*^+^/J) to prevent retinal degeneration.

### IOP measurement

IOP was measured using the microneedle method as described previously (John et al., 1998). Briefly, mice were anesthetized by intraperitoneal injection of a mixture of ketamine (99 mg/kg; Ketalar, Parke-Davis) and xylazine (9 mg/kg; Rompun, Phoenix Pharmaceutical). IOP was measured in both male and female mice and in intermixed small cohorts of C57BL/6J mice along with the experimental mice as a methodological control.

### Ocular biometry

We performed mouse ocular biometry using the Envisu R4300 spectral domain optical coherence tomography (SD-OCT; Leica/Bioptigen) as described previously (Park et al., 2012). Mice were anesthetized with ketamine/xylazine (100 mg/kg and 5 mg/kg, respectively) and their eyes dilated before performing ocular biometric measurements. The distance from the corneal surface to the retinal pigment epithelium/choroid interface provided axial length measurements. To minimize any confounding effect of body weight on ocular size, we only selected littermates with comparable body weights in each of the experimental groups.

### Ocular histology

Mice were euthanized and their eyes were enucleated and immersed in cold fixative (1% paraformaldehyde, 2% glutaraldehyde and 0.1 M cacodylate buffer) for a minimum of 48 h. Fixed eyes were transferred to 0.1 M cacodylate buffer solution and stored at 4°C until further use. The eyes were then embedded in glycol methacrylate and sectioned using a microtome. Serial sagittal sections (2 μm) were made by passing through the optic nerve. The sections were then stained with Hematoxylin and Eosin (H&E). H&E-stained ocular sections were assessed to determine the configuration of ocular angle structures. We evaluated at least 15-30 sections from central ocular regions sampled at regular intervals (10 µm). The distance between the peripheral retina and posterior edge of the Schlemm's canal was measured using ImageJ (National Institutes of Health) and the mean distance was calculated from a minimum of 10 central ocular sections/eye (4-6 eyes per group).

### Statistical analysis

Statistical comparisons between control and mutant groups or between multiple experimental groups at a given age were performed using two-tailed unpaired Student's *t*-test and one-way ANOVA, respectively, using the Prism version 6.0 software. A *P*-value of <0.05 was considered significant.

### Human study

#### Study approval

The present study screening for *PRSS56* variants in human subjects adhered to the tenets of the Declaration of Helsinki and was approved by the Institutional Review Board of the L V Prasad Eye Institute (LEC 08-15-097). Written informed consents were obtained from all adult subjects, whereas the legal guardians of the minors provided consent on their behalf.

#### Enrolment of patients and control subjects

Our study cohort comprised classical cases of PCG, along with their parents and normal relatives (*n*=957), who presented at the L V Prasad Eye Institute, Hyderabad, Southern India, between January 2001 and December 2017. Only those patients devoid of homozygous mutations in PCG-associated genes (*CYP1B1*, *LTBP2*, *TEK*, *MYOC* and *FOXC1*) were selected for further analysis (*n*=316). Ethnically-matched control subjects from the same geographical region as the patients (*n*=1291) were enrolled in this study. In addition, we also included a cohort of POAG patients (*n*=291) from the same geographical region. Detailed clinical inclusion and exclusion criteria for the enrolment of PCG, POAG and control subjects have been previously described (Kabra et al., 2017). Each subject was independently diagnosed by at least two glaucoma specialists and enrolled based on their inter-observer agreement. All individuals with a discordant diagnosis were excluded. The normal volunteers belonged to the longitudinal Andhra Pradesh Eye Disease Study (APEDS) cohort that has been followed up since April 1996. All the subjects underwent a comprehensive ocular examination as described earlier and did not manifest any signs or symptoms of glaucoma or any other ocular or systemic conditions (Kabra et al., 2017). The clinical parameters of the five PCG patients harboring the putative *PRSS56* mutations are provided in Table S3.

#### Targeted sequencing and validation

A pre-designed gene panel containing the entire coding and untranslated regions of the *PRSS56* gene was used for screening both the glaucoma cohorts (PCG and POAG) and normal controls by deep sequencing on an Ion Torrent platform using the Ion Ampliseq chemistry (Thermo Fisher Scientific). Measures of data generation and data capture based on our quality control parameters have been previously described (Kabra et al., 2017). The analysis pipeline included the stringent measures of filtering the reads followed by alignment of the obtained sequence data to the hg19 sequence. Samples that failed in the quality control measures were repeated. Further, all the *PRSS56* variants were validated by resequencing on an automated 3130xL DNA sequencer (Applied Biosystems) using the BigDye chemistry and by designing appropriate primers (Table S4).

#### Statistical analysis

The inter-observer agreements between two clinicians for the enrolment of subjects were based on kappa statistics. The allele frequencies of each variant were calculated by the gene counting method along with their odds ratios and 95% confidence intervals (Tables S1 and S2). The test of significance was calculated for the allele frequencies between cases and controls and a *P*-value of <0.05 was considered significant. A Bonferroni correction was carried out for the associated alleles. Hardy–Weinberg equilibrium (HWE) was estimated for the allele frequencies in controls. Alleles exhibiting deviations from HWE and monomorphic alleles were excluded from further analysis. The allele frequencies of the individual variations were compared to the overall frequencies provided at the 1000Genomes and gnomAD databases (Lek et al., 2016; The 1000 Genomes Project Consortium et al., 2010).

## Supplementary Material

Supplementary information
