# Supplementary material for: Loss of PRSS56 function leads to ocular angle defects and increased susceptibility to high intraocular pressure
Source: Dis Model Mech. 2020 May 29;13(5):dmm042853. doi: 10.1242/dmm.042853 (PMC7272341; doi:10.1242/dmm.042853)
Supplement: Supplementary information [file dmm-13-042853-s1.pdf]

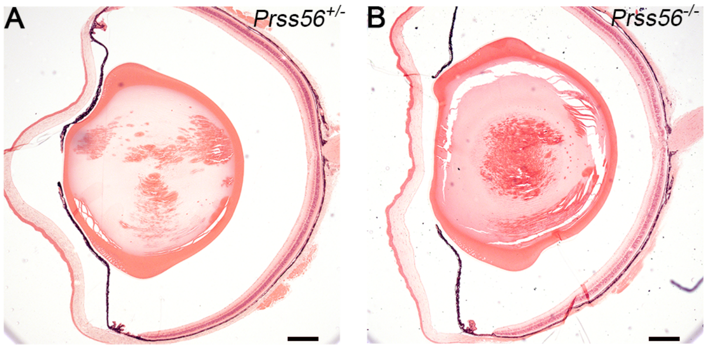

Figure S1. Histological analysis of whole *Prss56*$^{-/-}$ and *Prss56*$^{+/-}$ control eyes.
(A-B) Representative H&E stained ocular sections from *Prss56*$^{+/-}$ (A) and *Prss56*$^{-/-}$ mice (B) showing that *Prss56*$^{-/-}$ eyes do not exhibit gross morphological abnormalities compared to control *Prss56*$^{+/-}$ eyes. Scale bars=250μm.

Table S1. List of *PRSS56* variants identified in PCG cases and controls
Click here to download Table S1

Table S2. List of *PRSS56* variants identified in POAG cases and controls
Click here to download Table S2

Table S3. Clinical variables in PCG cases harboring *PRSS56* mutations
Click here to download Table S3

Table S4. List of primers used for sequencing *PRSS56* in patients and controls
Click here to download Table S4

Disease Models & Mechanisms • Supplementary information
